# Voltammetry Determination of Pb(II), Cd(II), and Zn(II) at Bismuth Film Electrode Combined with 8-Hydroxyquinoline as a Complexing Agent

**DOI:** 10.1155/2019/4593135

**Published:** 2019-07-03

**Authors:** Nguyen Mau Thanh, Nguyen Dinh Luyen, Tran Thanh Tam Toan, Nguyen Hai Phong, Nguyen Van Hop

**Affiliations:** ^1^University of Sciences, Hue University, Hue 530000, Vietnam; ^2^Faculty of Natural Sciences, Quang Binh University, Đồng Hới 510000, Vietnam; ^3^University of Education, Hue University, Hue 530000, Vietnam

## Abstract

A novel method was developed for the simultaneous determination of Pb(II), Cd(II), and Zn(II) based on the cathodic stripping response at a bismuth film electrode associated with oxine as a chelating agent. The developed method provided a high and sharp electrochemical response compared with the method without oxine. A linear response of peak currents was observed for Pb(II), Cd(II), and Zn(II) concentration in the range from 2 ppb to 110 ppb. The detection limits of Pb(II), Cd(II), and Zn(II) were 0.45, 0.17, and 0.78 ppb, respectively. This method was successfully applied to the determination of Pb(II), Cd(II), and Zn(II) in lake-water and river-water samples. The metals were detected at the ultratrace level, showing the feasibility of the proposed method for environmental applications.

## 1. Introduction

The release of different pollutants into the environment has increased significantly due to industrialisation. Among such pollutants, potentially toxic heavy metals, such as Pb(II), Cd(II), Hg(II), Ni(II), and Zn(II), are the most critical because they have a potentially damaging effect on human physiology and biological systems. Nevertheless, these metals have increasingly been used in industry in the production of anticorrosion coatings, pigments, alloys, and batteries [1]. Lead and cadmium are among the most toxic and hazardous. Lead is relatively harmful to human and living things [2]. Drinking water containing a high level of lead ions would cause serious disorders, such as nausea, convulsions, coma, renal failure, cancer, and subtle effects on the metabolism and intelligence [3]. Cadmium is accumulated in the human body, causing erythrocyte destruction, nausea, salivation, diarrhea, muscular cramps, renal degradation, and chronic pulmonary problems [4]. Zinc is considered as an essential trace element for human beings due to its relationship with the insulin production and because it plays the role of a catalyst for more than 200 enzymes [5]. An excessive consumption of Zn(II) (50 mg/day) can inhibit the absorption of copper (II) acquired from the human diet [6]. Therefore, the measurement of trace metal ions is very important for the environmental protection, food and agricultural chemistry, and high purity materials, and also for monitoring environmental pollution. Several sensitive techniques have been developed for the measurement of these metal ions. Flame atomic absorption spectrometry (FAAS) has widely been used for the determination of trace metal ions (Pb, Cd, and Cu) [7]. Biller and Bruland report the analysis of Mn, Fe, Co, Ni, Cu, Zn, Cd, and Pb in seawater using the Nobias-chelate PA1 resin and inductively coupled plasma mass spectrometry (ICP-MS) [8]. X-ray fluorescence analysis is employed to analyse Cu, Pb, As, Cd, Zn, Fe, Ni, and Mn [9]. These methods have excellent sensitivities and good selectivity; nevertheless several disadvantages such as long working time and high cost of instrument limit their applications.

Stripping voltammetry (SV) is a potential alternative for trace analyses due to numerous advantages such as faster analysis, higher selectivity and sensitivity, low cost, easy operation, and possibility to perform the analysis *in situ* [10, 11]. Mercury-based electrodes, such as mercury film electrodes and hanging mercury drop electrodes have traditionally been used in the stripping techniques due to their high sensitivity, reproducibility, purity of the surface, and possibility of amalgam formation. Hence, they have widely been accepted as the most sensitive electrodes for the determination of heavy metals [12]. However, the use of these techniques tends to decrease because the use of mercury affects the environment. Recently, bismuth film electrodes (BiFEs) have been developed as a successful alternative for “toxic” mercury electrodes and are, by now, widely recognised in a number of electroanalytical laboratories worldwide [13]. BiFEs have already been employed for the simultaneous or individual determination of many metal ions, such as Ni [14], Cr [15], Pb and Cd [16], and some organic compounds such as thiamethoxam [17], parathion [18], and others.

In order to enhance the selectivity and sensitivity of SV, several procedures have been developed in which SV is preceded by an adsorptive collection of complexed metals with specific chelating agents onto the electrode surface. Cu(II), Cd(II), and Pb(II) are determined by means of SV combined with oxine (8-hydroxyquinoline) as a chelating agent [19]. A number of trace metals in seawater are determined using cathodic stripping voltammetry with mixed 1igands (dimethylglyoxine and oxine) [20]. To the best of our knowledge, few papers have reported the simultaneous detection of Pb(II), Cd(II), and Zn(II) using stripping voltammetry at BiFE associated with the oxine ligand.

In the present paper, we extended the analytical utility of the bismuth film electrode with the development of a new method for the simultaneous determination of Pb(II), Cd(II), and Zn(II) using stripping voltammetry in combination with oxine as a complexing agent. The facial procedure that involved the *in situ* deposition of the bismuth film onto the glassy carbon electrode with a concurrent oxine-assisted accumulation of the analytes, followed by an cathodic stripping scan was demonstrated.

## 2. Experimental

### 2.1. Materials

All chemicals used in this study were of analytical reagent grade. Bismuth, lead, cadmium, and zinc standard stock solutions (1000 mg/L), sodium acetate (CH_3_COONa, 99%), acetic acid (CH_3_COOH, 99.8%), HEPES (2-[4-(2-hydroxyethyl)-1-piperazinyl]-ethanesulfonic acid), oxine (8-hydroxyquinoline), ammonia (NH_3_, 25%), sodium hydroxide (NaOH, 99%), and hydrochloric acid (HCl, 37%) were supplied from Merck company (Germany).

### 2.2. Apparatus

A three-electrode cell configuration was used for the voltammetric measurements. A glassy carbon electrode (2.8 mm diameter disk) was used as a working electrode, Ag/AgCl/KCl 3 M solution as a reference, and platinum wire as a counter electrode (CPA-HH5 Computerised Polarography Analyser, Vietnam).

### 2.3. Preparation of BiFE and Measurement Procedure

Before use, the glassy carbon electrode (GCE) was polished with 0.2 *μ*m alumina powder on a polishing pad. The electrode was then rinsed thoroughly with ethanol and dried naturally and ready to use. Prepare a solution of 0.1 M HEPES buffer solution containing Bi(III), Pb(II), Cd(II), Zn(II), and oxine, then a potentiostat switched on at an accumulation potential (*E*_acc.p_ = –1.6 V) (Figure S1(a)) for 240 s (Figure S1(b)) on the rotating glassy carbon disk electrode. During this step, Bi(III) was reduced to Bi to form an *in situ* Bi film on the GCE. The other metal ions (Zn, Pb, and Cd) were also reduced to corresponding metals depositing on GCE. Following the accumulation step, GCE stopped rotating, and the potentiostat switched on at the stripping and adsorption potential (*E*_str.p_ = –0.7 V) (Figure S1(c)) for 10 s (Figure S1(d)). The analyte metals were oxidised to corresponding metal ions. They immediately combined with the oxine ligand to form complexes on the electrode surface. Then, the voltammograms were recorded by applying a negative-going square-wave voltammetric potential scan prior to each measurement, and three “cleaning” scans at 0.3 V were carried out without the deposition step to completely remove the metal residues. The method is called square-wave adsorptive stripping voltammetry (SqW-AdSV).

### 2.4. Optimisation of Conditions for Preparing BiFE in Combination with Oxine

There are several factors affecting the electrochemical properties of BiFE. A primary study showed that the concentration of bismuth and oxine, as well as pH of the solution, significantly affected the electrochemical signals. In the present study, a Box–Behnken design (BBD) was applied to optimise the conditions for the *in situ* preparation of BiFE with the peak current in the determination of the metals as the response. The three variables (factors) with their experimental levels are shown in Table 1.

Based on the experimental data, a second-order polynomial model was obtained, which correlates the relationship between the responses and the studied variables. The relationship could be expressed as in equation (1).

(1) $$\begin{array}{c} y=b_{0}+b_{1}X_{1}+b_{2}X_{2}+b_{3}X_{3}+b_{11}X_{1}^{2}+b_{22}X_{2}^{2}+b_{33}X_{3}^{2}+b_{12}X_{1}X_{2}+b_{13}X_{1}X_{3}+b_{23}X_{2}X_{3}, \end{array}$$

where *y* is the predicted response value (peak current, *I*_p_, of Pb, Cd, and Zn); *X*_1_, *X*_2_, and *X*_3_ are the real independent variables (Table 1); *b*_0_ is the intercept term; *b*_1_, *b*_2_, and *b*_3_ are the linear coefficients; *b*_12_, *b*_13_, and *b*_23_ are the cross-product coefficients; and *b*_11_, *b*_22_, and *b*_33_ are the quadratic-term coefficients. All the coefficients of the equation from the experimental design were subjected to multiple nonlinear regression analyses by using the Minitab-16 software.

## 3. Results and Discussion

### 3.1. Preparing BiFE with Oxine as a Chelating Agent by BBD Approach

The traditional optimisation approach, that varies one variable at a time, is based on the experience that does not guarantee the attainment of the true optimum of the conditions for preparing *in situ* BiFE using oxine as a chelating agent. Conversely, the approach that relies on a rational experimental design, allowing the simultaneous variation of all the experimental factors, saves time and resources. Therefore, the experiments based on BBD were run in a random manner to minimise the effect of uncontrolled variables.  Table 2 shows the experimental design matrix and responses (cathodic peak current, *I*_pc_) derived from each experiment.

The response variables and independent variables (coded) are related following the second-order polynomial equations:

(2) $$\begin{array}{c} I_{\text{pc,Pb}}=1.95+0.08x_{1}+0.18x_{2}+0.12x_{3}-0.35x_{1}^{2}-0.44x_{2}^{2}-0.19x_{3}^{2}-0.00x_{1}x_{2}+0.00x_{1}x_{3}+0.11x_{2}x_{3}, \end{array}$$

(3) $$\begin{array}{c} I_{\text{pc,Cd}}=2.00+0.08x_{1}+0.22x_{2}+0.16x_{3}-0.37x_{1}^{2}-0.44x_{2}^{2}-0.18x_{3}^{2}-0.00x_{1}x_{2}-0.00x_{1}x_{3}+0.13x_{2}x_{3}, \end{array}$$

(4) $$\begin{array}{c} I_{\text{pc,Zn}}=2.00+0.08x_{1}+0.21x_{2}+0.15x_{3}-0.36x_{1}^{2}-0.45x_{2}^{2}-0.19x_{3}^{2}-0.00x_{1}x_{2}+0.00x_{1}x_{3}+0.11x_{2}x_{3}. \end{array}$$

The high values for the coefficient of determination (*R* ^2^) were found to be 0.994, 0.976, and 0.982 for Pb(II), Cd(II), and Zn(II), respectively, suggesting an excellent agreement between the experimental and estimated values. The positive and negative signs in each equation implied the positive and negative effect of the variables, respectively. The value of *b*_0_ reflects the average value of the peak current at the centre points (*b*_0_ = 1.95 *μ*A, 2.00 *μ*A, and 2.00 *μ*A for Pb, Cd, and Zn, respectively). The values of the coefficients indicated the amplitude of the effect. For clarity, the values and signs of the effect, as well as their *p* values of the three peak currents, are presented in Table 3. Interestingly, the influence pattern of the effect of all the three variables was very similar in terms of the values and the signs. All the linear and quadratic effects were statistically significant (*p* < 0.05). There were no interactions between Bi concentration and pH and the Bi concentration and oxine concentration for all the responses. As can be seen from the table, the intensity of the peak current for Pb was the lowest; therefore, the finding of conditions for achieving the maximal response of Pb was the focus of this study. The peak current of Pb had *p* ≤ 0.001, indicating that the model was compatible with the experimental data. Furthermore, the coefficient of determination (*R* ^2^) was calculated as 0.995, indicating that the predictive model was able to explain 99.5% of the variability of the response. No significant lack of fit was obtained (*p*=0.294). The results suggested that the estimated model was significant and adequate to represent the relationship between *I*_pc,Pb_ and the Bi concentration, pH, and oxine concentration.

**Figure 1:**
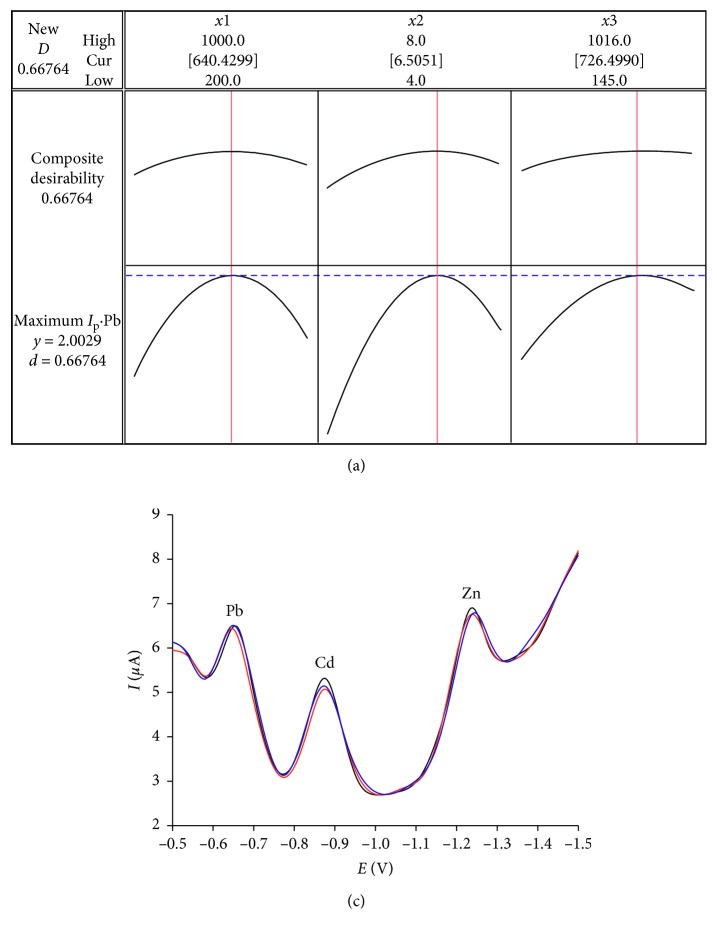
Profiles for predicated values (a) and reliability function for peak current of Pb (b).

The profile for predictive values in Minitab-16 was used for the optimisation process. The optimisation design matrix (Figure 1(a)) represents the maximal *I*_p_ (2.0029 *μ*A for Pb) at the variable set as follows: bismuth concentration (*X*_1_): 640 ppb; pH of solution (*X*_2_): 6.5; oxine concentration (*X*_3_): 726 ppb. The reliability of this prediction was examined by conducting three similar experiments at the optimal conditions. The *I*_p_ values obtained were 2.162 *μ*A, 1.834 *μ*A, and 1.867 *μ*A. The one-sample *t*-test showed a nonsignificant difference with the respective values presented by the model (*t*(2) = –0.47, *p*=0.69). These optimal conditions were used to prepare the Bi-film electrode in the research.  Figure 1(b) shows the SqW-AdSV curves of Pb(II), Cd(II), and Zn(II) reduction at the optimal conditions. The peak-to-peak separation of Pb-Cd and Cd-Zn were calculated as 0.22 V and 0.36 V, respectively. The reasonable separation suggested that the simultaneous determination of the analytes was possible.

### 3.2. Electrochemical Behavior of Analytes at BiFE Using Oxine as a Chelating Agent

#### 3.2.1. Electrochemical Behavior at Different Electrodes

**Figure 2:**
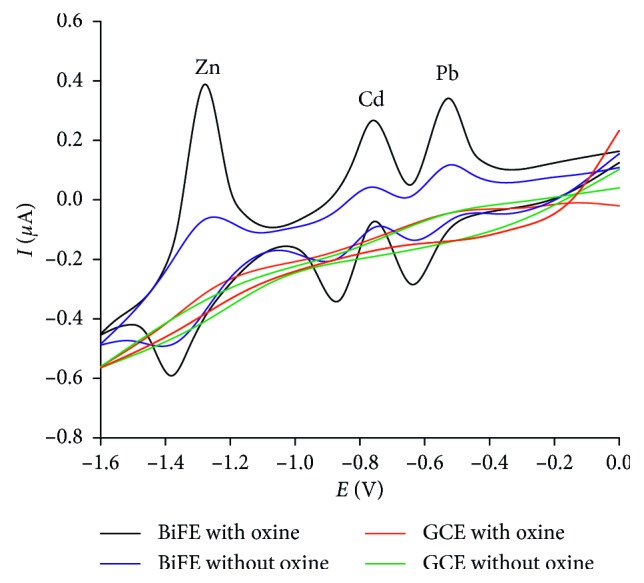
Cyclic voltammetric curves of Pb(II), Cd(II), and Zn(II) at GCE and BiFE with and without oxine (10 ppb Pb, 10 ppb Zn, and 5 ppb Cd in HEPES buffer pH 6).

The electrochemical behavior of Pb(II), Cd(II), and Zn(II) in the HEPES buffer solution of pH 6.5 with and without oxine was studied using cyclic voltammetry (CV) in the potential range from 0 to 1.6 V.  Figure 2 shows that broad and low signs were observed on bare GCE both with and without oxine (green and red lines). However, the CV responses for these three analytes at BiFE were clearly observed (blue line). It was worth noting that adding oxine to the studied solution increased significantly the peak currents of the analytes. The peak current were higher significantly than those without oxine (3.8 times for Pb, 4.6 times for Cd, 5.1 times for Zn at anodic peak current and 3.4 times for Pb, 2.9 times for Cd, 2.3 times for Zn at cathodic peak). 8-hydroxyquinoline, also known as 8-quinolinol or oxine, is a chelating agent. It contains an oxygen donor atom and a nitrogen donor atom that can bind to metal ions to form complexes. Such complexes adsorbed and accumulated on the working electrode. The increments of peak currents can be contributed to the accumulation of the metal complexes on the electrode surface.

#### 3.2.2. Effects of pH

Because protons participate in the electrode reaction of Pb, Cd, and Zn, the effect of pH on the voltammetric behavior of Pb, Cd, and Zn at BiFE was studied by means of SqW-AdSV in the pH range of 4.2–7.8 (under the conditions: 10 ppb Pb(II), 10 ppb Zn(II), and 5 ppb Cd(II); bismuth concentration: 640 ppb; oxine concentration: 726 ppb, accumulation potential: −1.6 V, accumulation time: 250 s, stripping and adsorptive potential: −0.7 V, stripping time: 10 s, cathodic scan rate: 0.2 Vs^−1^). It was found that the cathodic peak potentials (*E*_pc_) of the three analytes shifted to more negative values (roughly from −50 to −220 mV) with the increase of pH (from 4.2 to 7.8). The relationship between the peak potential (*E*_pc_, V) and pH exhibited a high linearity (equations (5)–(7)).

(5) $$\begin{array}{c} E_{\text{pc,Zn}}=\left(-1.037\pm 0.009\right)+\left(-0.050\pm 0.006\right)\text{pH,}\;r=0.993, \end{array}$$

(6) $$\begin{array}{c} E_{\text{pc,Cd}}=\left(-0.480\pm 0.040\right)+\left(-0.065\pm 0.007\right)\text{pH},\;r=0.995, \end{array}$$

(7) $$\begin{array}{c} E_{\text{pc,Pb}}=\left(-0.270\pm 0.030\right)+\left(-0.062\pm 0.004\right)\text{pH,}\;r=0.995. \end{array}$$

These slopes of –0.050, –0.065, and –0.062 for Zn, Cd, and Pb, respectively, were closed to the theoretical value of –0.059/pH at 25°C expected from the Nernst equation. This indicates that the electrochemical process of each analyte took place with an equal number of protons and electrons.

#### 3.2.3. Effects of Scan Rate

Some information concerning the electrochemical mechanism can be provided from the relationship between the voltammetric signs and the scan rates (denoted as *v*). In fact, the effect of scan rate on the electrochemical response of 10 ppb Pb(II), 10 ppb Zn(II), and 5 ppb Cd(II) on BiFE was investigated using CV with the scan rate ranging from 0.2 V·s^−1^ to 0.4 V·s^−1^ in the HEPES buffer solution with pH 6.5 and the presence of 640 ppb Bi(III) and 726 ppb oxine. The linear plots of cathodic peak current (*I*_pc_) against the square root of the scan rate (*v* ^1/2^) were drawn to determine whether the electroreductive reaction was controlled by adsorption or diffusion. If the plot of *I*_pc_ *versusv* ^1/2^ is linear and passes the origin, this process is controlled by diffusion [21]. The linear regression equations of *I*_pc_ (*μ*A) for Pb, Cd, and Zn oxidation *versusv* ^1/2^ are as as follows:

(8) $$\begin{array}{c} I_{\text{pc,Pb}}=\left(-0.7\pm 1.4\right)+\left(7.1\pm 2.6\right)v^{1/2},\;r=0.980, \end{array}$$

(9) $$\begin{array}{c} I_{\text{pc,Cd }}=\left(-0.8\pm 1.2\right)+\left(7.2\pm 2.2\right)v^{1/2},\;r=0.986, \end{array}$$

(10) $$\begin{array}{c} I_{\text{pc,Zn}}=\left(-0.5\pm 1.1\right)+\left(7.3\pm 2.1\right)v^{1/2},\;r=0.989. \end{array}$$

The plots of *I*_p_ *versusv* ^1/2^ were highly linear (*r* = 0.980–0.989, *p* < 0.05). The number after plus and minus is the 95% confidence interval. For Pb, Cd, and Zn, the intercept passed the origin because the 95% confidence interval for this parameter contained 0 (varying from –2.1 to 0.7 for Pb, –2.0 to 0.4 for Cd and –1.6 to 0.5 for Zn). This indicates that the electrode process of the electroreduction of the analytes was controlled by diffusion.

Based on the Laviron theory [22], the relationship between the peak potential (*E*_pc_) and the natural logarithm of the scan rates is described as follows:

(11) $$\begin{array}{c} E_{\text{p}}=E^{0}+\frac{RT}{\left(1-\alpha \right)nF}\ln\frac{\left(1-\alpha \right)nFk_{\text{s}}}{RT}-\frac{RT}{\left(1-\alpha \right)nF}\ln\;v, \end{array}$$

where *α* is the charge transfer coefficient, *k*_s_ is the heterogeneous electron transfer rate constant of the surface-confined redox couple, *n* is the number of electrons transferred, *v* is the scan rate (V·s^−1^), and *E* ^0^ is the formal redox potential, *T* = 298 K, *R* = 8.314 J·mol·K^−1^, and *F* = 96480 C·mol^−1^.

The plots of *E*_pc_ (V) of the analytes *versus*ln *v* were linear form and the linear regression equations are as follows:

(12) $$\begin{array}{c} E_{\text{pc,Pb}}=\left(-0.708\pm 0.005\right)+\left(-0.023\pm 0.005\right)\ln\;v,\;r=0.939, \end{array}$$

(13) $$\begin{array}{c} E_{\text{pc,Cd}}=\left(-0.920\pm 0.010\right)+\left(-0.026\pm 0.010\right)\ln\;v,\;r=0.828, \end{array}$$

(14) $$\begin{array}{c} E_{\text{pc,Zn}}=\left(-1.429\pm 0.006\right)+\left(-0.028\pm 0.005\right)\cdot \ln\;v,\;r=0.952. \end{array}$$

**Figure 3:**
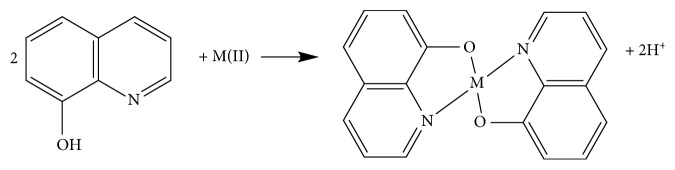
Complexing reaction between metal (M) and oxine.

The values of (1 − *α*) · *n* for Pb, Cd and Zn can be obtained from the slope of equations (12)–(14), and they were 1.12, 0.99, and 0.92, respectively. The value of *α* is assumed to be equal to 0.5 [23]. Therefore, the number of electrons transferred (*n*) in the electroreduction of Pb(II), Cd(II), and Zn(II) was 2.24, 1.98, and 1.84, respectively. Consequently, assuming *n* = 2, the oxidation mechanism for Pb, Cd, and Zn could involve two electrons and two protons. Oxine is characterised by the presence of a N,O-binding system that is suitable for interacting strongly with Zn(II), Cd(II), and Pb(II) ions. It is assumed that Zn(II), Cd(II), and Pb(II) ions could be connected to two oxines to form metal complex (M(oxine)_2_) [24] (Figure 3).

M(II) is Pb(II), Cd(II), and Zn(II). The reactions at BiFE in aqueous solutions were proposed as follows:

(a) The accumulation step at −1.6 V
  (15) $$\begin{array}{c} \text{Bi}\left(\text{III}\right)+3\text{HOx}⟶\text{Bi}{\left(\text{Ox}\right)}_{3}+{3\text{H}}^{+}\\ \text{M}\left(\text{II}\right)+2\text{HOx}⟶\text{M}{\left(\text{Ox}\right)}_{2}+{2\text{H}}^{+} \end{array}$$
(i) where M is Pb(II), Cd(II) and Zn(II); HOx is oxine.
  (16) $$\begin{array}{c} \text{Bi}{\left(\text{Ox}\right)}_{3}+3\text{e}+{3\text{H}}^{+}⟶\text{Bi}\left(\text{0}\right)/\text{GCE}\left(\text{denoted as BiFE}\right)+3\text{HOx}\\ \text{M}{\left(\text{Ox}\right)}_{2}+2\text{e}+{2\text{H}}^{+}⟶\text{M}\left(\text{0}\right)/\text{BiFE}+2\text{HOx} \end{array}$$
(b) The stripping and adsorption step at BiFE at −0.7 V
  (17) $$\begin{array}{c} \text{M}\left(\text{0}\right)/\text{BiFE}-2\text{e}+2\text{HOx}⟶\text{M}{\left(\text{Ox}\right)}_{2\left(\text{adsorption}\right)}/\text{BiFE}+{2\text{H}}^{+} \end{array}$$
(c) Square-wave voltammetric potential scan is applied for loading metals to BiFE. The reduction process involved two electrons and two protons as follows:
  (18) $$\begin{array}{c} \text{M}{\left(\text{Ox}\right)}_{2\left(\text{adsorption}\right)}/\text{BiFE}+{2\text{H}}^{+}+2\text{e}⟶\text{M}\left(\text{0}\right)/\text{BiFE}+2\text{HOx} \end{array}$$
(d) Electrode surface cleaning step:
  (19) $$\begin{array}{c} \text{M}\left(\text{0}\right)/\text{BiFE}-5\text{e}⟶\text{M}\left(\text{II}\right)+\text{Bi}\left(\text{III}\right) \end{array}$$

#### 3.2.4. Effects of Interferents

The interference of other substances on the electrochemical response of BiFE in the detection of Pb, Cd, and Zn was studied under the optimal conditions. Na_2_SO_4_, KHCO_3_, CaCl_2_, Mg(NO_3_)_2_, Cu(NO_3_)_2_, and Co(CH_3_COO)_2_ were chosen as interferents because they were commonly found with the target analytes in lake and river water. The relative error (RE) was taken as the relative deviation of the peak current measured with and without interferents. These substances did not show any interference with Pb(II), Zn(II), and Cd(II) detection (RE < 5%) (Tables S2–S4). However, Cu(II) and Co(II) significantly affect the electrochemical signals when their concentration is greater than 10 and 15 times, respectively (Tables S5-S6) (RE = 15.8% as Cu(II)/analyte (ppb/ppb) = 10, RE = 10% as Co(II)/analyte (ppb/ppb) = 15). These results suggested that the proposed method has a relevant selectivity towards the simultaneous determination of Pb(II), Cd(II), and Zn(II) in water in lakes and rivers.

### 3.3. Repeatability, Linear Range, Limit of Detection (LOD), and Reproducibility

#### 3.3.1. Repeatability

The SqW-AdSV replicate measurements at BiFE was performed with different analyte concentrations. The SqW-AdSV curves were measured in the mixtures of the same concentration of Pb, Cd, and Zn at 10 ppb, 20 ppb, and 30 ppb. Each SqW-AdSV signal was obtained by successive measurements for eight times (Figure S2). The obtained RSD for Pb, Cd, and Zn was lower than 10.6%, 8.4%, and 5.9% for 10 ppb, 20 ppb, and 30 ppb levels, respectively. These values were lower than a half of RSD calculated from the Horwitz equation (½ RSD_H_) [25]. Such reasonable RSD of the successive measurements indicated that the developed method could be repeatedly employed for the detection of Pb, Cd, and Zn in either low or high concentration range.

#### 3.3.2. Linear Range and Limit of Detection (LOD)

**Figure 4:**
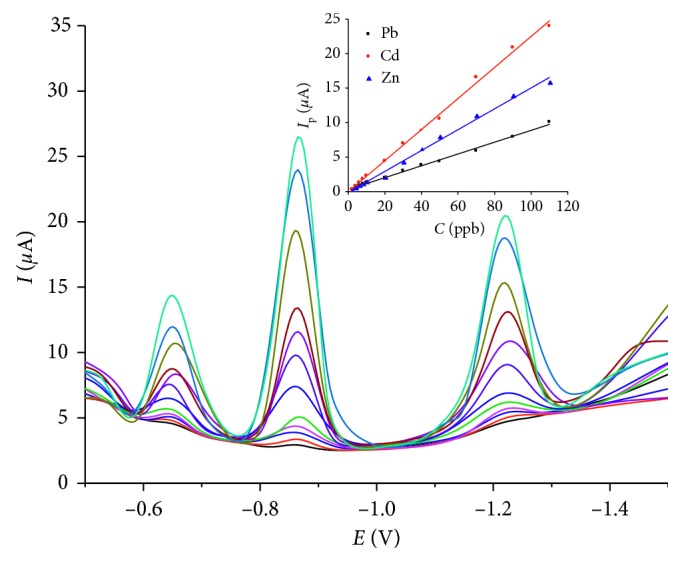
SqW-AdSV voltammograms using BiFE under optimal conditions in solutions containing different concentrations of Pb, Cd, and Zn (2–110 ppb each metal). Upper small figure is a linear regression line for Cd (red), Pb (blue), and Zn (black) (conditions: 0.01 M HEPES buffer solution pH 6.5; [oxine] = 726 ppb; [Bi(III)] = 640 ppb; *E*_acc.p_ = –1.6 V; *t*_acc.p_ = 240 s; *E*_str.p_ = –0.7 V; *t*_str.p_ = 15 s; potential scanning range *E*_range_ = –0.35 ÷ –1.5 V; square-wave amplitude Δ*E* = 0.05 V; potential step *U*_step_ = 0.006 V; increment time *t*_step_ = 0.3 s; measuring time *t*_meas_ = 3 ms; electrode rotating rate *ω* = 2000 rpm).

(i) For the individual determination of the target analytes, the concentration of one of them varied from 2 to 110 ppb while keeping the other two constant at 20 ppb (data not shown). The cathodic peak current of each of the metal increased linearly with its concentrations (*C*) in the range from 2 to 110 ppb, while the signals of the other two remained constant. The linear regression equations for Pb, Cd, and Zn were *I*_p_ = (0.290 ± 0.090) · *C*_Pb_ + (0.086 ± 0.002), (*R* ^2^ = 0.996); *I*_p_ = (–0.040 ± 0.200) · *C*_Cd_ + (0.225 ± 0.004), (*R* ^2^ = 0.997); and *I*_p_ = (–0.100 ± 0.200) · *C*_Zn_ + (0.151 ± 0.004), (*R* ^2^ = 0.993), respectively. Based on 3*S*_b_/*m* with *S*_b_ being the residual standard deviation of the peak current (*I*_p_) and *m* the slope of the calibration plot, the limit of detection (LOD) was calculated as 0.40 ppb for Pb(II), 0.13 ppb for Cd(II), and 0.46 ppb for Zn(II).
(ii) In the case of simultaneous increase in the concentrations of the target analytes, the SqW-AdSV voltammograms obtained at BiFE with oxine in HEPES buffer solution pH 6.5 are shown in Figure 4. As can be seen from the figure, the peak current of Pb, Cd, and Zn increased linearly with increasing concentrations over the range from 2 to 110 ppb for Pb, Cd, and Zn. The linear regression equations are as follows:
  (20) $$\begin{array}{c} I_{\text{pc,Zn}}=\left(0.883\pm 0.094\right)+\left(0.228\pm 0.004\right)C_{\text{Zn}},\;r=0.986, \end{array}$$
  (21) $$\begin{array}{c} I_{\text{pc,Cd}}=\left(-0.530\pm 0.089\right)+\left(0.228\pm 0.004\right)C_{\text{Cd}},\;r=0.999, \end{array}$$
  (22) $$\begin{array}{c} I_{\text{pc,Pb}}=\left(-0.835\pm 0.109\right)+\left(0.178\pm 0.005\right)C_{\text{Pb}},\;r=0.998. \end{array}$$

The LOD for the simultaneous measurement was 0.45, 0.17, and 0.78 ppb for Pb, Cd, and Zn, respectively. It is worth noting that this LOD for each species was nearly equal to that with the individual measurement (0.40 ppb for Pb(II), 0.13 ppb for Cd(II), 0.46 ppb for Zn(II)), indicating that the analytes did not interfere with each other in the determination. Compared with other methods, the developed one here was advantageous in terms of low detection limit and compatible linear range (Table 4).

#### 3.3.3. Reproducibility

Reproducibility of the electrochemical response is of special interest for automatic monitoring of potentially toxic heavy metals. Hence, the response of BiFE was performed for a ten-day period by immersing the electrode in a solution of spiked water with 10 ppb Pb, 10 ppb Cd and 10 ppb Zn (10 measurements were performed during the working-day period). The electrode was stored in the spiked solution between each analysis at a potential of −0.15 V. The changes of average *I*_p_ *versus* time was insignificant (*p* < 0.05). The RSDs of *I*_p_ for Pb, Cd and Zn were 9.3, 8.8 and 5.5%, respectively using the same electrode for all the measurements. These values were lower than ½ RSD_H_ [25], indicating proposed SqW-AdSV method exhibited the high reproducibility.

### 3.4. Analysis of Real Samples

**Figure 5:**
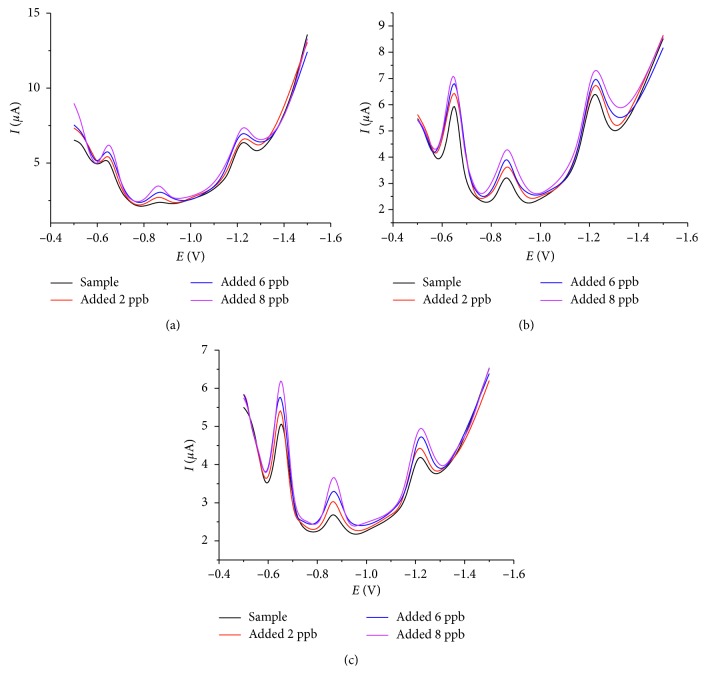
SqW-AdSV voltammograms obtained for the water samples: (a) Cau Rao River, (b) Nam Ly Lake, and (c) Kien Giang River (the same conditions as in Figure 4).

The water from a lake and two rivers in Quang Binh province, Vietnam, namely, Cau Rao River, Nam Ly Lake, and Kien Giang River, was used to determine the concentration of lead, cadmium, and zinc using the proposed method (SqW-AdSV) and GF-AAS for the sake of comparison. The concentrations of the target metal are listed in Table 5. The paired sample *t*-test showed that there were no significant differences between the two methods (Pb: *t*(2) = 0.04, *p*=0.97; Cd: *t*(2) = 0.59, *p*=0.62; Zn: *t*(2) = 0.38, *p*=0.74). The recovery test rate was 94% to 107% for Pb, 96% to 105% for Cd, and 92% to 104% for Zn, suggesting that there were no important matrix interferences for the samples analysed by the proposed SqW-AdSV method. As can be observed, the three metals were detected at the ultra-trace level showing the feasibility of the proposed method for environmental applications.  Figure 5 shows the SqW-AdSV voltammograms obtained for the water samples.

## 4. Conclusions

Using Box–Behnken design allowed optimising the preparation of BiFE with oxine as a chelating agent. The resulting electrode was successfully used for the simultaneous determination of Pb(II), Cd(II), and Zn(II) with a low detection limit, wide linear range, and good selectivity and without the interference of each other. A satisfied recovery and reproducibility of the proposed method were also obtained. This method was successfully applied to the determination of Pb(II), Cd(II), and Zn(II) in real water samples from lakes and rivers.

## Figures and Tables

**Table 1:** Factors in BBD and their levels.

	Bismuth concentration (*X*_1_, ppb)	pH (*X*_2_)	Oxine concentration (*X*_3_, ppb)
Central level (0)	600	6	581
High level (+1)	1000	8	1016
Low level (–1)	200	4	145

**Table 2:** Design matrix and responses for full factorial design.

Runs	Coded variable levels			Peak current *I*_pc_ (*μ*A)		
	*x*_1_	*x*_2_	*x*_3_	Pb	Cd	Zn
1	0	0	0	1.92 ± 0.31^a^	1.97 ± 0.44	1.96 ± 0.34
2	0	+1	+1	1.78 ± 0.52	1.99 ± 0.32	1.92 ± 0.31
3	–1	–1	0	0.91 ± 0.41	0.94 ± 0.33	0.93 ± 0.52
4	0	–1	–1	1.08 ± 0.25	1.02 ± 0.41	1.01 ± 0.11
5	0	0	0	1.95 ± 0.35	2.00 ± 0.15	2.00 ± 0.11
6	–1	0	–1	1.23 ± 0.41	1.26 ± 0.25	1.26 ± 0.21
7	0	–1	+1	1.16 ± 0.15	1.19 ± 0.17	1.18 ± 0.41
8	+1	0	–1	1.39 ± 0.25	1.43 ± 0.22	1.43 ± 0.25
9	–1	0	+1	1.43 ± 0.35	1.47 ± 0.38	1.46 ± 0.35
10	0	+1	–1	1.28 ± 0.32	1.32 ± 0.26	1.31 ± 0.32
11	+1	–1	0	1.07 ± 0.31	1.10 ± 0.31	1.10 ± 0.28
12	+1	+1	0	1.40 ± 0.42	1.44 ± 0.42	1.43 ± 0.43
13	0	0	0	1.98 ± 0.38	2.04 ± 0.44	2.03 ± 0.11
14	–1	+1	0	1.25 ± 0.26	1.29 ± 0.20	1.28 ± 0.18
15	+1	0	+1	1.59 ± 0.18	1.64 ± 0.35	1.63 ± 0.24

^a^Peak current is expressed as mean ± standard deviation (*n* = 3).

**Table 3:** Analysis of variance (ANOVA) for B-B design.

Terms	Coefficient *I*_pc,_ _Pb_	*p*	Coefficient *I*_pc,_ _Cd_	*p*	Coefficient *I*_pc,_ _Zn_	*p*
Constant	1.95	≤0.001	2.00	≤0.001	2.00	≤0.001
*x*_1_	0.08	0.003	0.08	0.061	0.08	0.042
*x*_2_	0.19	≤0.001	0.22	0.001	0.21	0.001
*x*_3_	0.12	0.001	0.16	0.006	0.15	0.005
*x*_1_^2^	–0.35	≤0.001	–0.37	0.001	–0.36	≤0.001
*x*_2_^2^	–0.44	≤0.001	–0.44	≤0.001	–0.45	≤0.001
*x*_3_^2^	–0.19	≤0.001	–0.18	0.014	–0.19	0.008
*x*_1_ · *x*_2_	0.00	0.909	0.00	0.960	0.00	0.912
*x*_1_ · *x*_3_	0.00	1.000	0.00	1.000	0.00	1.000
*x*_2_ · *x*_3_	0.11	0.004	0.13	0.047	0.11	0.050
Regression		≤0.001				
Lack of fit		0.294				

**Table 4:** Comparison of LOD and linear range related to different electrodes and methods for determination of analytes.

Electrode	Method	Analyte	LOD (ppb)	Linear range (ppb)	Ref.
Hg-Bi/SWNTs/GCE	SqW-AdSV	Cd(II)	0.98	10–130	[26]
		Pb(II)	1.3		
		Zn(II)	2		

BiFEs	SqW-AdSV	Cd(II)	0.82	5.6–39.2	[27]
		Pb(II)	1.64	4.1–41.4	
		Zn(II)	0.08	2.6–39.2	

G/PANI/NME	SqW-AdSV	Cd(II)	0.1	1–300	[28]
		Pb(II)	0.1	1–300	
		Zn(II)	1.0	1–300	

Ƴ-AlOOH@SiO2/Fe3O4/GCE	SqW-AdSV	Zn(II)	2.6	2616–36624	[29]
		Cd(II)	1.12	1120–15680	
		Pb(II)	0.4	414–99456	

Bi/GCE	SqW-AdSV	Cd(II)	0.49	0.05–100	[30]
		Pb(II)	0.41	0.05–100	

BiFE	SqW-AdSV	Zn(II)	0.78	2–110	This work
		Cd(II)	0.17	2–110	
		Pb(II)	0.45	2–110	

SWNT: single-walled carbon nanotube; G/PANI/NME: graphene-based polyaniline nanocomposite-modified electrode.

**Table 5:** Pb, Cd, and Zn concentrations in real samples analysed by SqW-AdSV and GF-AAS.

Notation	SqW-AdSV			GF-AAS		
	Pb (ppb)	Cd (ppb)	Zn (ppb)	Pb (ppb)	Cd (ppb)	Zn (ppb)
Cau Rao River	15.0 ± 8.2^a^	4.3 ± 0.7	13.6 ± 4.0	16.4 ± 4.7	3.9 ± 0.6	12.8 ± 3.6
Nam Ly Lake	26.9 ± 5.1	5.1 ± 2.4	20.3 ± 3.8	27.1 ± 2.1	4.6 ± 0.3	18.3 ± 2.0
Kien Giang River	21.7 ± 4.4	3.1 ± 1.5	7.3 ± 2.6	20.0 ± 5.3	3.5 ± 0.7	8.9 ± 1.7

^a^Data are expressed as mean ± SD (standard deviation) (*n* = 3).

## Data Availability

The data used to support the findings of this study are included within the article and within the supplementary information file.

## Supplementary Materials

Supplementary Materials Figure S1: effects of operational parameters. Figure S2: successive measurements of SqW-AdSV curves for eight times. Table S1: effects of Na_2_SO_4_ on the stripping peak current. Table S2: effects of KHCO_3_ on the stripping peak current. Table S3: effects of CaCl_2_ on the stripping peak current. Table S4: effects of Mg(NO_3_)_2_ on the stripping peak current. Table S5: effects of Co(CH_3_COO)_2_ on the stripping peak current. Table S6: effects of Cu(NO_3_)_2_ on the stripping peak current.
