# Supplementary material for: Voltammetry Determination of Pb(II), Cd(II), and Zn(II) at Bismuth Film Electrode Combined with 8-Hydroxyquinoline as a Complexing Agent
Source: J Anal Methods Chem. 2019 Jul 3;2019:4593135. doi: 10.1155/2019/4593135 (PMC6636451; doi:10.1155/2019/4593135)
Supplement: Supplementary Materials — Figure S1: effects of operational parameters. Figure S2: successive measurements of SqW-AdSV curves for eight times. Table S1: effects of Na2SO4 on the stripping peak current. Table S2: effects of KHCO3 on the stripping peak current. Table S3: effects of CaCl2 on the stripping peak current. Table S4: effects of Mg(NO3)2 on the stripping peak current. Table S5: effects of Co(CH3COO)2 on the stripping peak current. Table S6: effects of Cu(NO3)2 on the stripping peak current. [file 4593135.f1.pdf]

Supplementary materials

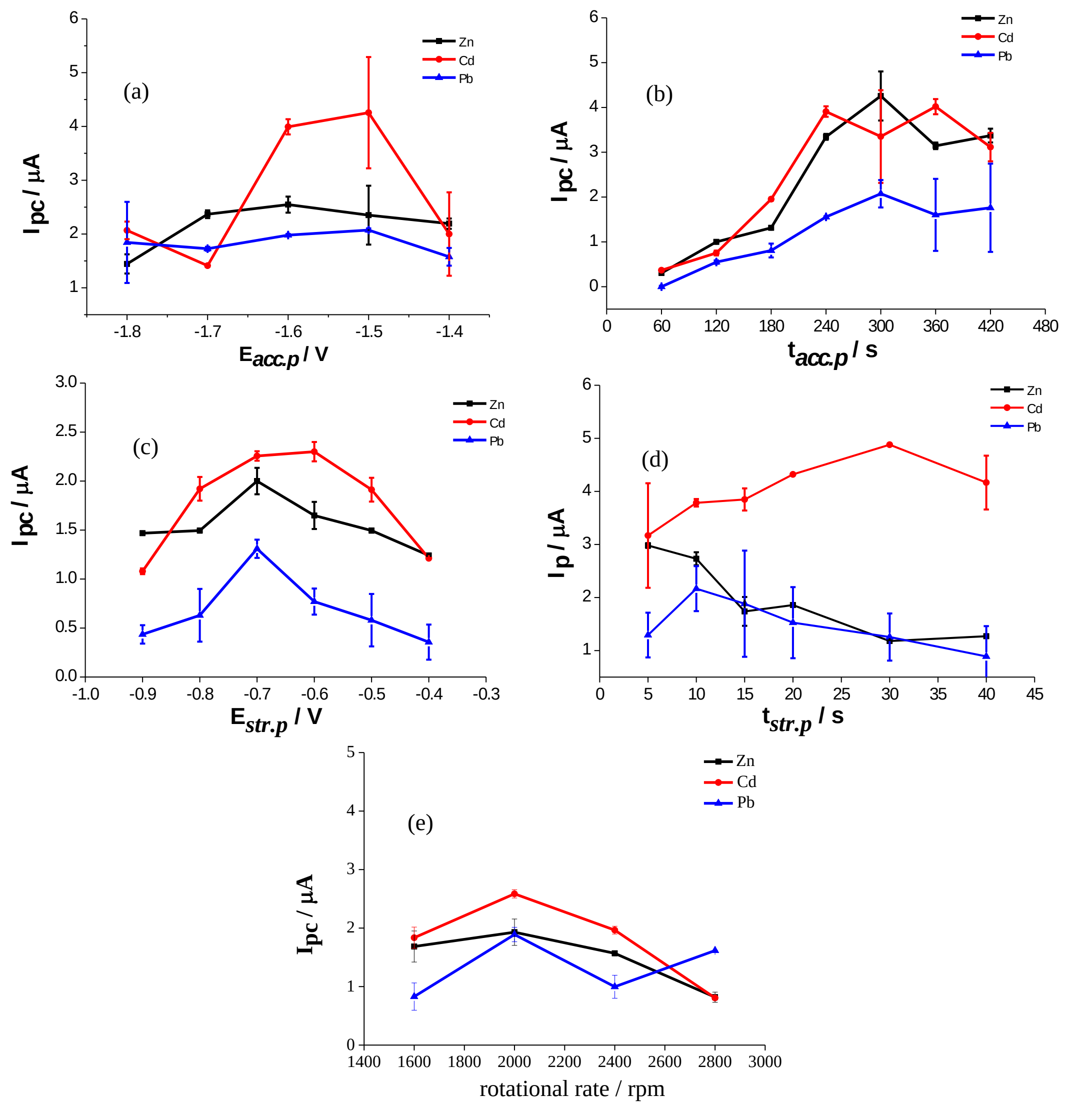

Figure S1. Effect of operational parameters: (a) accumulation potential, (b) accumulation time, (c) stripping potential, (d) stripping time, (e) and rotational rate of WE. on cathodic peak current of Pb, Cd, and Zn

Figures S1 (a, b) show the effect of accumulation potential and time on the cathodic peak currents ($I_{pc,Pb}$, $I_{pc,Cd}$, $I_{pc,Zn}$). As can be seen from the figures, the peak current of cadmium and lead peaked at –1.5 V, but at this potential the peak current was less stable (large error deviation) than that at –1.6 V. The cathodic peak current of zinc peaked at -1.6 V and decreased slightly when the accumulation potential increased further. Generally, the accumulation potential greater than -1.6 V, the peak current tended to be unstable (large error bar). Therefore, the value of –1.6 V was chosen as the accumulation potential. The cathodic peak currents increased

practically linearly with the time up to 240 s, and after that it rose unstably, therefore, 240 s was chosen as the time for the accumulation of the metals (Figure 1S(b)). The cathodic peak currents of the analytes (Pb, Cd, Zn) were found to peak at the stripping potential of –0.7 V (Figure 1S(c)). The stripping time of 10s was selected for compromising high and stable peak current (Figure 1S(d)). The peak current was also studied with the rotation rate of the electrode, The rotation rate of 2,000 rpm was relevant for the process (Figure 1S(e)).

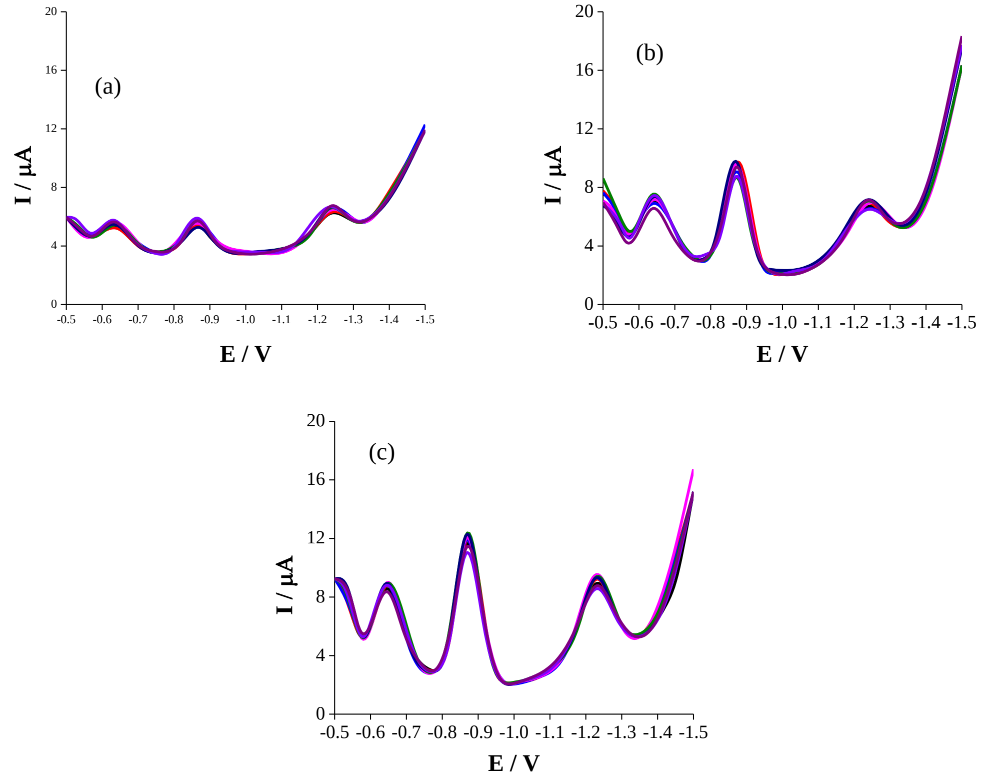

Figure S2. Successive measurements of SqW–AdSV curves for eight times: a) $C_{Pb} = C_{Cd} = C_{Zn}$ = 10 ppb; b) $C_{Pb} = C_{Cd} = C_{Zn}$ = 20 ppb; c) $C_{Pb} = C_{Cd} = C_{Zn}$ = 30 ppb in 0.01 M HEPES pH 6.5.

The SqW–AdSV curves were measured repeatedly eight times at equal concentrations of Pb(II), Cd(II) and Zn(II) (Figure S2).

The relative error (*RE*) was calculated based on the expression

$$RE\ (\%) = \frac{I_{p,c} - I_0}{I_0} \times 100$$

where $I_{p,c}$ and I_0 are the peak current of the analyte measured with and without an interferent, respectively.

RE (%) was lower than 5 % (Table S1–S4) even the concentration of the interferents was 100 times higher than the concentration of the substance to be analysed.

Table S1. Effect of Na_2SO_4 on the stripping peak current

Interferent/ analyte (ppb/ppb)	Zn		Cd		Pb	
	I_p	*RE* (%)	I_p	*RE* (%)	I_p	*RE* (%)
0 : 1	2.132	0.0	2.024	0.0	1.342	0.0
10 : 1	2.083	–2.3	1.993	–1.5	1.403	4.5
20 : 1	2.041	–4.3	1.989	–1.7	1.306	–2.7
40 : 1	2.045	–4.1	2.001	–1.1	1.279	–4.7
70 : 1	2.042	–4.2	1.941	–4.1	1.300	–3.1
100 : 1	2.033	–4.6	1.963	–3.0	1.285	–4.2

Table S2. Effect of $KHCO_3$ on the stripping peak current

Interferent/ analyte (ppb/ppb)	Zn		Cd		Pb	
	I_p	*RE* (%)	I_p	*RE* (%)	I_p	*RE* (%)
0 : 1	2.205	0.0	2.131	0.0	1.392	0.0
10 : 1	2.313	4.9	2.194	3.0	1.403	0.8
20 : 1	2.295	4.1	2.103	–1.3	1.410	1.3
40 : 1	2.108	–4.4	2.029	–4.8	1.403	0.8
70 : 1	2.172	–1.5	2.114	–0.8	1.359	–2.4
100 : 1	2.277	3.3	2.031	–4.7	1.336	–4.0

Table S3. Effect of $CaCl_2$ on the stripping peak current

Interferent : analyte (ppb/ppb)	Zn		Cd		Pb	
	I_p	*RE* (%)	I_p	*RE* (%)	I_p	*RE* (%)
0 : 1	2.175	0.0	2.144	0.0	1.392	0.0
10 : 1	2.136	–1.8	2.125	–0.9	–0.9	1.6

20 : 1	2.273	4.5	2.167	1.1	1.452	4.3
40 : 1	2.215	1.8	2.131	–0.6	1.358	–2.5
70 : 1	2.108	–3.1	2.103	–1.9	1.347	–3.2
100 : 1	2.117	–2.7	2.076	–3.2	1.363	–2.1

Table S4. Effect of $Mg(NO_3)_2$ on the stripping peak current

Interferent/ analyte (ppb/ppb)	Zn		Cd		Pb	
	I_p	*RE* (%)	I_p	*RE* (%)	I_p	*RE* (%)
0 : 1	2.254	0.0	2.166	0.0	1.390	0.0
10 : 1	2.178	–3.4	2.138	–1.3	1.363	–1.9
20 : 1	2.206	–2.1	2.105	–2.8	1.413	1.7
40 : 1	2.308	2.4	2.216	2.3	1.382	–0.6
70 : 1	2.315	2.7	2.203	1.7	1.407	1.2
100 : 1	2.327	3.2	2.156	–0.5	1.415	1.8

Table S5. Effect of $Co(CH_3COO)_2$ on the stripping peak current

Interferent/ analyte (ppb/ppb)	Zn		Cd		Pb	
	I_p	*RE* (%)	I_p	*RE* (%)	I_p	*RE* (%)
0 : 1	2.213	0.0	2.329	0.0	1.319	0.0
5 : 1	2.154	-2.7	2.301	-1.2	1.375	4.2
10 : 1	2.104	-4.9	2.219	-4.7	1.381	4.7
15 : 1	2.041	-7.8	2.050	-12.0	1.451	10.0
20 : 1	2.009	-9.2	1.973	-15.3	1.534	16.3
30 : 1	1.623	-26.7	1.775	-23.8	1.528	15.8

Table S6. Effect of $Cu(NO_3)_2$ on the stripping peak current

Interferent : analyte (ppb/ppb)	Zn		Cd		Pb	
	I_p	*RE* (%)	I_p	*RE* (%)	I_p	*RE* (%)
0 : 1	2.141	0.0	2.237	0.0	1.367	0.0
5 : 1	2.098	-2.0	2.159	-3.5	1.411	3.2

10 : 1	1.943	-9.2	2.008	-10.2	1.583	15.8
15 : 1	1.717	-19.8	2.090	-6.6	1.665	21.8
20 : 1	1.587	-25.9	1.625	-27.4	1.872	36.9
30 : 1	1.506	-29.7	1.755	-21.6	2.070	51.4
